# Medical Lexicon, or Modern Terminology

**Published:** 1845-09

**Authors:** 


*Medical Lexicon, or Modern Terminology,* being a complete vocabulary of definitions, including all the technical terms employed by writers and teachers of Medical Science at the present day, and comprising several hundreds of words not found in any other Dictionary. Designed for the use of students and practitioners. By David Meredith Reese, A. M., M. D., late Professor of the Institutes of Medicine and Surgery, and Medical Jurisprudence, in the Washington University of Baltimore, &c., &c. pp. 240, 24mo. New York: H. G. Langley, 1845.

To the medical student especially, the above work is invaluable. The want of such a dictionary has long been felt, and in its preparation, Dr. Reese has rendered an important service to all who are engaged in the study of medicine. It will also be found very useful and convenient to the practitioner. The work being designed as a Pocket Companion to students, the author has brought it within the narrowest possible limits, avoiding all reference to the etymology of terms, he has confined himself, in every case, to the briefest definition that could be given. For the origin and derivation of words, other Lexicons will still be necessary; but for definitions simply, which is all that it professes to give, this work will answer all the purposes of a larger dictionary.

